# Coculture of *Bifidobacterium bifidum* G9‐1 With Butyrate‐Producing Bacteria Promotes Butyrate Production

**DOI:** 10.1111/1348-0421.13224

**Published:** 2025-04-23

**Authors:** Haruka Yokota, Yoshiki Tanaka, Hiroshi Ohno

**Affiliations:** ^1^ Biofermin Pharmaceutical Co. Ltd. Kobe Japan

**Keywords:** acetate, *Bifidobacterium bifidum* G9‐1 (BBG9‐1), butyrate‐producing bacteria, coculture, lactate, probiotics, short‐chain fatty acids (SCFA)

## Abstract

Supplementation with *Bifidobacterium bifidum* G9‐1 (BBG9‐1) has been established to enhance the production of butyrate, a short‐chain fatty acid (SCFA) known for its beneficial effects in alleviating constipation. We hypothesized that BBG9‐1 alters gut microbiota such that bacteria that produce butyric acid from lactate and acetate become more abundant. In this study, we sought to determine whether BBG9‐1 promotes the growth of butyrate‐producing bacteria and thereby enhances butyrate production. BBG9‐1 was cocultured with different butyrate‐producing bacteria to compare differences in the SCFA production of cocultures and monocultures. We indeed detected significant increases in the production of SCFAs in cocultures compared to monocultures. Moreover, lactate and butyrate production increased in a time‐dependent manner in the BBG9‐1 and *Faecalibacterium prausnitzii* ID 6052 coculture. In addition, acetate production in cocultures initially increased until 16 h, followed by a decline between 20 and 24 h, and a subsequent significant increase at 48 h. Comparatively, lactate and acetate production in the BBG9‐1 and *Anaerostipes caccae* JCM 13470^T^ coculture peaked at 16 h and declined thereafter, and butyrate production increased in a time‐dependent manner. In contrast, lactate, acetate, and butyrate production in the BBG9‐1 and *Roseburia hominis* JCM 17582^T^ coculture increased in a time‐dependent manner. These findings indicate that butyrate‐producing bacteria increase butyrate production by utilizing BBG9‐1‐produced lactate and acetate. Thus, the butyrate‐mediated physiological activity of BBG9‐1 could be attributed to an indirect enhancement of butyrate production.

**Abbreviations**
BBG9‐1: *Bifidobacterium bifidum* G9‐1
FOS: fructooligosaccharides
GAM: Gifu Anaerobic Medium
GOS: galactooligosaccharides
HPLC: high‐performance liquid chromatography
OD: optical density
RCM: reinforced clostridial medium
SCFA(s): short‐chain fatty acid(s)
YC: yeast extract and casitone
YCFA: yeast extract, casitone, and fatty acid

## 1 Introduction

Originating from the word “probiosis,” which implies coexistence, probiotics are defined as live microorganisms that confer beneficial effects in hosts when consumed in adequate amounts [1]. Although probiotic intake has been associated with improvements in a range of different health conditions, including a lowering of blood pressure, plasma insulin, and body mass index [2], the mechanisms underlying the effects of these probiotics on the host and gut microbiota have yet to be fully understood. Accordingly, it is important to elucidate these mechanisms to enable the safe and effective application of probiotics.

*Bifidobacterium bifidum* G9‐1 (BBG9‐1) is the active constituent of intestinal regulators such as BIOFERMIN TABLETS and BIOFERMIN BIFIDUS POWDER (Biofermin Pharmaceutical Co. Ltd., Kobe, Japan), which are available as prescription drugs, and has been established to be effective in alleviating constipation [3, 4, 5] and diarrhea [6]. It has also been found that supplementing the gut microbiota with BBG9‐1 promotes butyrate production and the expression of Tph‐1 [4], thereby enhancing the production of serotonin, a neurotransmitter. Furthermore, BBG9‐1 may contribute to promoting intestinal motility and alleviating constipation [4, 7]. However, given that BBG9‐1 lacks an intrinsic capacity to produce butyrate, the mechanisms by which this *Bifidobacterium* strain enhances butyrate production remain to be elucidated.

Short‐chain fatty acids (SCFAs), including butyrate, are products of the fermentation of dietary fiber by the gut microbiota. They are saturated aliphatic organic acids consisting of one to six carbons, with acetate and butyrate being abundant in the large intestines and feces [8]. Acetate may prevent pathogenic infection by promoting the protection of host epithelial cells [9]. In addition, acetate may alleviate constipation by promoting an increase in stool water content, thus enhancing intestinal motility, and by promoting serotonin production in the colon [10]. Butyrate has been shown to suppress the symptoms of inflammatory diseases, such as inflammatory bowel disease, via homeostatic and anti‐inflammatory activity within the intestinal epithelium [11]. SCFAs absorbed in the colon are transported into the superior or inferior mesenteric vein, which both drain into the portal vein and liver. Interestingly, some SCFAs can also bypass the liver via the pelvic plexus, which drains into the inferior vena cava, thereby reaching the systemic circulation to directly affect substrate metabolism and the function of peripheral tissues [12].

Peripheral SCFAs stimulate GPR41 and GPR43 and induce intracellular signaling by activating or inhibiting heterotrimeric G proteins and enzymes, contributing to host health, including alleviation of obesity, diabetes, inflammation, and cardiovascular disease [13].

*Anaerostipes caccae* (*A. caccae*), *Faecalibacterium prausnitzii* (*F. prausnitzii*), and *Roseburia hominis* (*R. hominis*) are butyrate‐producing bacteria found in the human intestines. Butyrate, acetate, and lactate are the main products of glucose metabolism in *A. caccae* [14], whereas *F. prausnitzii* produces butyrate, formate, and lactate [15], and *R. hominis* produces butyrate and formate [16]. Moreover, it has also been established that the butyrate produced by these bacteria is derived from the lactate and acetate produced by *Bifidobacteria* [17, 18].

We hypothesized that acetate produced by BBG9‐1 promotes the growth of butyrate‐producing bacteria in the intestines, consequently enhancing butyrate production. In this study, we thus aimed to elucidate the mechanisms by which BBG9‐1 promotes butyrate production in the intestines.

## 2 Materials and Methods

### 2.1 Bacteria

BBG9‐1 (Biofermin Pharmaceutical Co. Ltd., Kobe, Japan) was cultured with Aneromate‐J (Shimadzu Diagnostics Corporation, Tokyo, Japan) for 18 h under anaerobic conditions at 37°C using a medium comprising AccuDia Gifu Anaerobic Medium (GAM) Broth (Shimadzu Diagnostics Corporation) supplemented with 0.7% glucose (FUJIFILM Wako Pure Chemical Corporation, Osaka, Japan) and 0.1% polysorbate 80 (JUNSEI CHEMICAL Co. Ltd., Tokyo, Japan; modified GAM). Following cultivation, bacterial cells were harvested by centrifuging cultures at 14,000 *g* for 5 min and stored at −80°C until used for further analyses.

*F. prausnitzii* ID 6052 (Biofermin Pharmaceutical Co. Ltd.), *A. caccae* JCM 13470^T^ (RIKEN BRC, Tsukuba, Japan), and *R. hominis* JCM 17582^T^ (RIKEN BRC) were used as butyrate‐producing bacteria; they were cultured for 24 h under anaerobic conditions at 37°C using reinforced clostridial medium (RCM), modified GAM, and brain heart infusion broth (KANTO CHEMICAL CO. INC., Tokyo, Japan), respectively. YC + FOS medium was used for *F. prausnitzii*, whereas YC + GOS medium was used for *A. caccae* and *R. hominis*. After cultivation, bacterial cells were harvested by centrifugation and stored at −80°C for further use. The RCM used was prepared by adding 10.0 g of Bacto Peptone (Thermo Fisher Scientific Inc., Waltham, MA, USA), 10.0 g of Difco Beef extract (Thermo Fisher Scientific Inc.), 3.0 g of yeast extract (Thermo Fisher Scientific Inc.), 5.0 g of glucose (FUJIFILM Wako Pure Chemical Corporation), 5.0 g of sodium chloride (FUJIFILM Wako Pure Chemical Corporation), 0.5 g of l‐cysteine hydrochloride hydrate (FUJIFILM Wako Pure Chemical Corporation), and 1.0 g of sodium acetate (FUJIFILM Wako Pure Chemical Corporation) to 1 L of purified water.

### 2.2 Preparation of Coculture Media

In this study, coculture media were prepared by adding 3.5 g/L galactooligosaccharides (GOS, FUJIFILM Wako Pure Chemical Corporation) or fructooligosaccharides (FOS, FUJIFILM Wako Pure Chemical Corporation) to yeast extract, casitone, and fatty acid (YCFA) medium [19] without SCFAs (YC medium). The media were adjusted to a pH of 7.0 ± 0.2, autoclaved at 121°C for 15 min, and preliminarily reduced under anaerobic conditions (nitrogen:carbon dioxide:hydrogen = 80:10:10) for > 12 h in an anaerobic incubator (TE‐HER ANAEROBOX ANX‐3; HIRASAWA Co. Ltd., Tokyo, Japan). The medium to which GOS was added was YC + GOS, whereas that to which FOS was added was YC + FOS.

### 2.3 Coculture

BBG9‐1 and each of the aforementioned butyrate‐producing bacteria (*F. prausnitzii*, *A. caccae*, and *R. hominis*) were used to inoculate YC + GOS or YC + FOS medium to a final concentration of 1.0 × 10^6^ cfu/mL and cultured under anaerobic conditions at 37°C for 48 h. In total, 80 mL of coculture media were transferred into 100‐mL vials. Samples of culture media (1.5 mL) were collected at 0, 8, 12, 16, 20, 24, and 48 h after the start of culturing, and the turbidity (OD 600 nm), pH, and organic acid contents of the culture supernatants were then measured using a spectrophotometer (Tecan Group Ltd., Männedorf, Switzerland), a pH meter (HORIBA Ltd., Kyoto, Japan), and a high‐performance liquid chromatograph (details on this method are provided in the subsequent section), respectively. The culture supernatants used were obtained by centrifugation at 14,000 *g* and 4°C for 5 min. YC + FOS medium was used for *F. prausnitzii*, whereas YC + GOS medium was used for *A. caccae* and *R. hominis*. As experimental groups, we used the following cultures: BBG9‐1 monoculture, monocultures of the three butyrate‐producing bacteria, and cocultures of BBG9‐1 with each of the three butyrate‐producing bacteria (*n* = 6/group).

### 2.4 High‐Performance Liquid Chromatography

Samples for high‐performance liquid chromatography (HPLC) analysis were prepared by filtering ~1 mL of culture supernatant using 0.2‐µm membrane filters (Merck Millipore, Darmstadt, Germany). SCFAs were then quantified using an HPLC system comprising a Shimadzu organic acid analysis system (Shimadzu Corporation, Kyoto, Japan), an electrical conductivity detector (CDD‐10A, Shimadzu Corporation), and a Shim‐pack SCR‐102(H) column (300 × 8 mm ID, Shimadzu Corporation). The separation medium used was 5 mmol/L *p*‐toluenesulfonic acid, which was delivered at a flow rate of 0.8 mL/min at 50°C [20]. The following SCFAs were determined: lactate, acetate, and butyrate. Each SCFA standard was accurately weighed to 1000 mg/L, transferred to a 100‐mL volumetric flask, and dissolved with ultrapure water. SCFA levels were determined from the area under the peak using a calibration curve.

### 2.5 Statistical Analyses

Data are presented as the means ± standard error (means ± S.E.). Comparisons between the butyrate‐producing bacterial monocultures and the corresponding coculture groups were subjected to statistical analysis using Welch's *t*‐test in Excel, with the level of significance being set at 5% (two‐sided).

## 3 Results

### 3.1 Coculture of BBG9‐1 and *F. prausnitzii*

**Figure 1:**
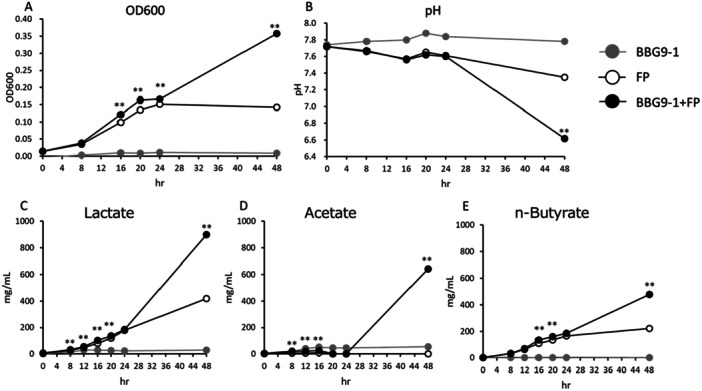
Changes in OD_600_, pH, and short‐chain fatty acid production in mono‐ and cocultures of BBG9‐1 and *Faecalibacterium prausnitzii* cultivated in YC + FOS medium. (A) OD_600_. (B) pH. (C) Lactate. (D) Acetate. (E) *n*‐Butyrate. Data are presented as the means ± standard error (means ± S.E. *n* = 6). **p < 0.01, as determined using Welch's *t*‐test. BBG9‐1, *Bifidobacterium bifidum* G9‐1 monoculture; *F. prausnitzii*, *F. prausnitzii* ID 6052 monoculture; BBG9‐1 + *F. prausnitzii*, coculture of *B. bifidum* G9‐1 and *F. prausnitzii* ID 6052.

The OD_600_ value of the BBG9‐1 + *F. prausnitzii* culture medium was significantly higher than that of the *F. prausnitzii* monoculture medium after 16, 20, 24, and 48 h of culture (Figure 1A), whereas the pH of the BBG9‐1 + *F. prausnitzii* culture medium was significantly lower than that of the *F. prausnitzii* monoculture medium at 48 h (Figure 1B). Quantification of SCFAs in the culture supernatants revealed significant increases in lactate production in the BBG9‐1 + *F. prausnitzii* coculture after 8, 12, 16, 20, and 48 h of culture, compared with that in the *F. prausnitzii* monoculture, with the increase being time‐dependent (Figure 1C). Similarly, compared with that in the *F. prausnitzii* monoculture, acetate production in the BBG9‐1 + *F. prausnitzii* coculture increased significantly after 8, 12, 16, and 48 h of culture (Figure 1D). Acetate production in the BBG9‐1 + *F. prausnitzii* coculture initially increased until 16 h of culture, and then declined between 20 and 24 h, although it subsequently increased again at 48 h (Figure 1D). Moreover, compared with that in the *F. prausnitzii* monoculture, we detected a significant increase in *n*‐butyrate production in the BBG9‐1 + *F. prausnitzii* coculture after 16, 20, and 48 h of culture, and similar to lactate, the increase in *n*‐butyrate production was time‐dependent (Figure 1E).

### 3.2 Coculture of BBG9‐1 and *A. caccae*

**Figure 2:**
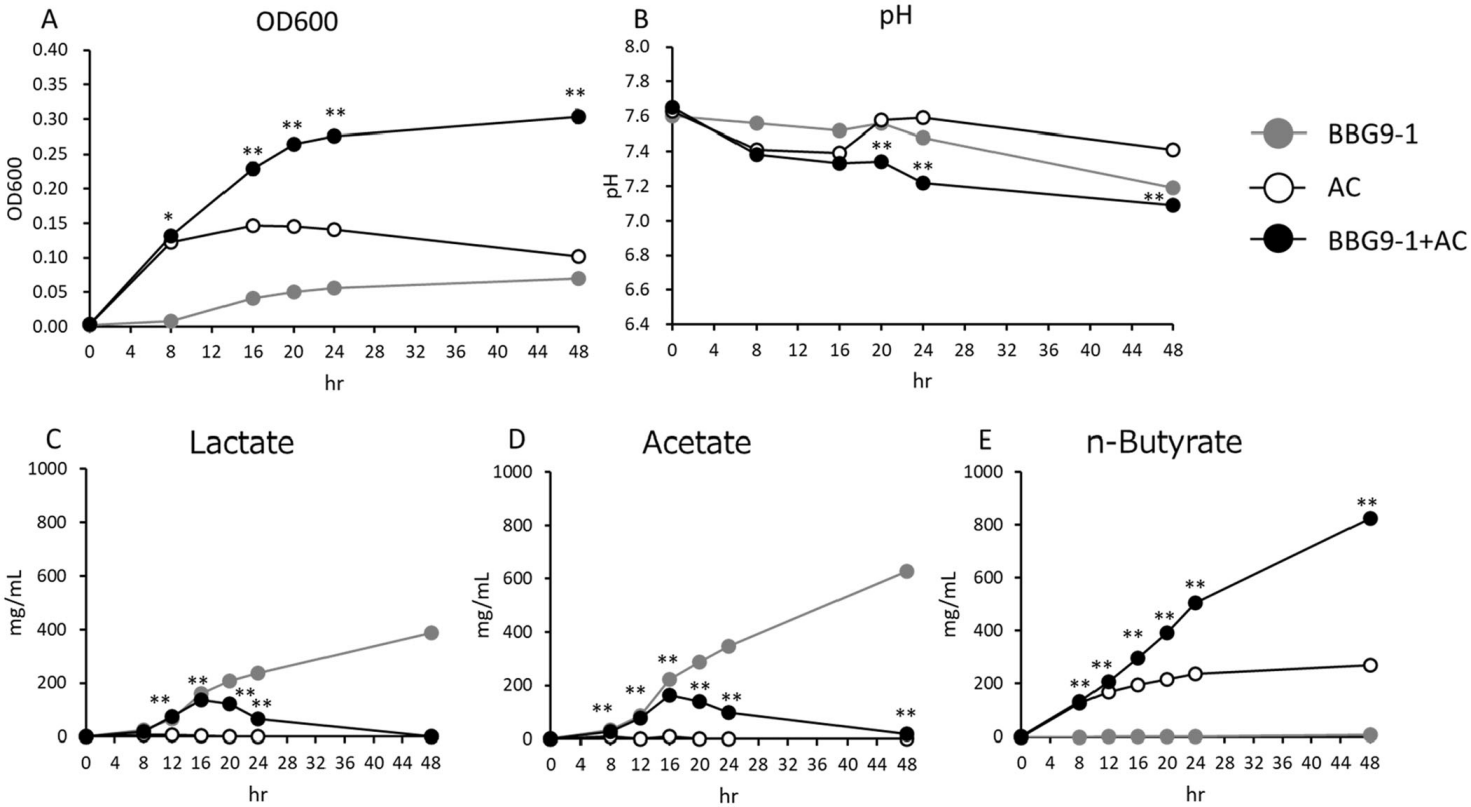
Changes in OD_600_, pH, and short‐chain fatty acid production in mono‐ and cocultures of BBG9‐1 and *Anaerostipes caccae* cultivated in YC + GOS medium. (A) OD_600_. (B) pH. (C) Lactate. (D) Acetate. (E) *n*‐Butyrate. Data are presented as the means ± S.E. (*n* = 6). **p < 0.01, *p < 0.05, as determined using Welch's *t*‐test. BBG9‐1, *Bifidobacterium bifidum* G9‐1 monoculture; *A. caccae*, *A. caccae* JCM 13470^T^ monoculture; BBG9‐1 + *A. caccae*, coculture of *B. bifidum* G9‐1 and *A. caccae* JCM 13470^T^.

When measured after culturing for 8, 16, 20, 24, and 48 h, the OD_600_ of the BBG9‐1 + *A. caccae* culture medium was significantly higher than that of the *A. caccae* culture medium (Figure 2A), whereas the pH of the BBG9‐1 + *A. caccae* culture medium was significantly lower than that of the *A. caccae* culture medium after 20, 24, and 48 h of culture (Figure 2B). Compared with that in the *A. caccae* monoculture, lactate production in the BBG9‐1 + *A. caccae* coculture increased significantly after culturing for 12, 16, 20, and 24 h (Figure 2C). Lactate production in the BBG9‐1 + *A. caccae* coculture peaked after culturing for 16 h and declined thereafter (Figure 2C). Compared with that in the *A. caccae* monoculture, acetate production increased significantly in the BBG9‐1 + *A. caccae* coculture after 8, 12, 16, 20, 24, and 48 h of culture (Figure 2D). Similar to that of lactate, the production of acetate in BBG9‐1 + *A. caccae* peaked after culturing for 16 h and declined thereafter (Figure 2D). In addition, significant time‐dependent increases were noted for *n*‐butyrate production after culturing for 8, 12, 16, 20, 24, and 48 h (Figure 2E).

### 3.3 Coculture of BBG9‐1 and *R. hominis*

**Figure 3:**
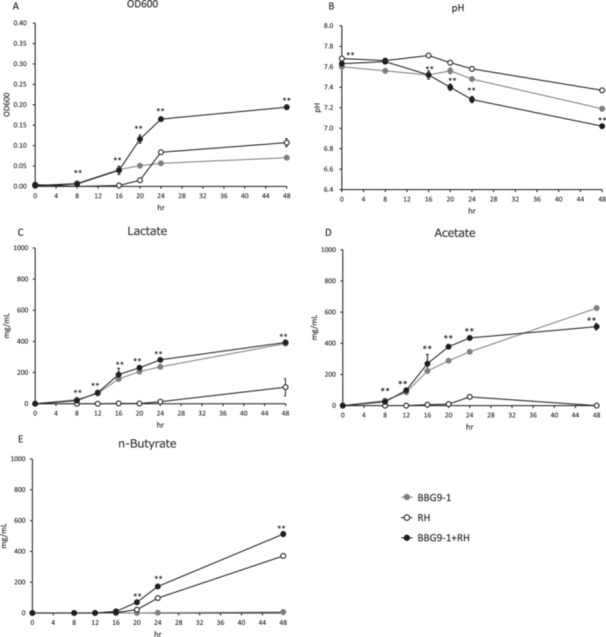
Changes in OD_600_, pH, and short‐chain fatty acid production in mono‐ and cocultures of BBG9‐1 and *Roseburia hominis* cultivated in YC + GOS medium. (A) OD_600_. (B) pH. (C) Lactate. (D) Acetate. (E) *n*‐Butyrate. Data are presented as the means ± S.E. (*n* = 6). **p < 0.01, as determined using Welch's *t*‐test. *Bifidobacterium bifidum* G9‐1 monoculture; *R. hominis*, *R. hominis* JCM 17582^T^ in monoculture; BBG9‐1 + *R. hominis*, cocultures of *B. bifidum* G9‐1 and *R. hominis* JCM 17582^T^.

After culturing for 8, 16, 20, 24, and 48 h, the OD_600_ of the BBG9‐1 + *R. hominis* coculture medium was significantly higher than that of the *R. hominis* monoculture medium (Figure 3A), whereas the pH of the BBG9‐1 + *R. hominis* coculture medium was significantly lower than that of the *R. hominis* monoculture medium after culturing for 16, 20, 24, and 48 h (Figure 3B). Significant increases in the lactate and acetate production in the BBG9‐1 + *R. hominis* coculture were detected after culturing for 8, 12, 16, 20, 24, and 48 h. In the case of *n*‐butyrate, significant time‐dependent increases in production were detected after culturing for 20, 24, and 48 h, compared with that in the *R. hominis* monoculture (Figure 3C–E).

## 4 Discussion

The SCFAs produced by the gut microbiota may contribute to alleviating constipation and various gastrointestinal and metabolic disorders [7, 10, 21]. Supplementing the microbiota with the probiotic BBG9‐1 has previously been found to enhance butyrate production [4]. Although BBG9‐1 does not produce butyrate, BBG9‐1‐induced changes in the gut environment may produce conditions that are conducive to the production of butyrate by butyrate‐producing bacteria. Consequently, in this study, we used cocultures of BBG9‐1 and selected butyrate‐producing bacteria to investigate whether BBG9‐1 promotes the growth of these butyrate producers and thus increases butyrate production.

*F. prausnitzii* is a gram‐negative obligate anaerobe that has been identified as the most abundant bacterium in the gut microbiota of healthy human adults [22, 23]. In terms of SCFAs, it mainly produces butyrate, lactate, and formate, and requires acetate for growth [15]. In addition, its anti‐inflammatory activity and association with the alleviation of inflammatory bowel disease and obesity have been elucidated [24]. In a previous study, *F. prausnitzii* S3L/3 and *B. adolescentis* L2‐32 were cocultured on FOS and YC media, and acetate production 24 h after incubation was decreased compared to that at 8 h, while butyrate production was increased [25]. These findings indicate that *F. prausnitzii* uses acetate to produce butyrate. In the present study, compared with that in the *F. prausnitzii* monoculture, we detected significant increases in the production of lactate, acetate, and *n*‐butyrate in the BBG9‐1 and *F. prausnitzii* coculture (Figure 1C–E). The observed fluctuations in acetate production (Figure 1D) indicate that *F. prausnitzii* produces butyrate by utilizing the acetate produced by *Bifidobacteria* (Figure 1E). However, the medium and culture conditions assessed in this study resulted in poor BBG9‐1 growth and low acetate production (Figure 1A,D). Consequently, while an increase in butyrate production during coculture may be attributed to the utilization of BBG9‐1‐produced acetate, it could also be ascribed to the promotion of *F. prausnitzii* growth by bacterial components or other metabolites of BBG9‐1. In preliminary tests, growth was confirmed in *F. prausnitzii* and BBG9‐1 using GOS and FOS, respectively; *F. prausnitzii* grew less efficiently on GOS than on FOS, while BBG9‐1 grew on both GOS and FOS, so only FOS was selected for *F. prausnitzii* and BBG9‐1 coculture. However, *F. prausnitzii* grew poorly in general, possibly due in part to the slightly higher pH at inoculation for FOS than for GOS, which was not a suitable pH for the growth of BBG9‐1. Similarly, culture of *Bifidobacterium bifidum* (*B. bifidum*) in a medium comprising FOS and YCFA resulted in poor growth [17, 25]. Given that *B. bifidum* tends to be inefficient in the degradation and assimilation of FOS, the medium and culture conditions used in this study may have led to poor growth. Further studies examining the effects of the inoculation amount, medium components, and medium pH at the time of inoculation may enable identification of the factor(s) contributing to this poor growth.

*A. caccae* is a gram‐positive obligate anaerobic bacterium that utilizes acetate and lactate and mainly produces butyrate, acetate, and lactate [14, 17], and may contribute to protecting against allergic reactions to food [26]. It also has the capacity to produce vitamin B_12_ [27], which plays an important role in cellular metabolism, particularly in DNA synthesis, methylation, and mitochondrial function [28]. In the present study, compared with the monoculture of *A. caccae*, we detected significant increases in lactate, acetate, and *n*‐butyrate production in the coculture of BBG9‐1 and *A. caccae* (Figure 2C–E). The production of lactate and acetate increased from the start of culture, peaked at 16 h, and declined thereafter (Figure 2C,D). These findings indicate that *A. caccae* produces butyrate by utilizing the lactate and acetate produced by the cocultured *Bifidobacterium* (Figure 2E).

*R. hominis* is a gram‐negative to gram‐variable, slightly curved, obligate anaerobe that mainly produces butyrate and formate [16]; it is associated with the alleviation of ulcerative colitis [29] and neuroinflammation [20]. Lactate is a by‐product of glucose fermentation [16], and *R. hominis* requires acetate [16] for growth [30]. In the present study, compared with that in the *R. hominis* monoculture, we detected significant increases in the production of lactate, acetate, and *n*‐butyrate in the coculture of BBG9‐1 and *R. hominis* (Figure 3C–E). The production of acetate increased in a time‐dependent manner although the slope of acetate production was smaller in the coculture than in the BBG9‐1 monoculture after 36 h of culture (Figure 3D). On the basis of these observations, we speculate that a proportion of the acetate produced by BBG9‐1 was utilized by *R. hominis*. In contrast to the trends in lactate and acetate production using *A. caccae* (Figure 2C,D), we failed to observe a comparable initial increase in production in *R. hominis* until 16 h of culture, followed by a decline in *R. hominis*. In the *R. hominis* BBG9‐1 monoculture, the growth phase was between 8 and 24 h of culture (Figure 3A), and acetate production increased accordingly (Figure 3D). In contrast, the *R. hominis* monoculture was characterized by slower growth than the BBG9‐1 monoculture, with a growth phase between 16 and 24 h of culture. This could be associated with a mismatch between the rate of acetate production by BBG9‐1 and the rate of its subsequent conversion to butyrate. Alternatively, this could be attributed to an inability to utilize acetate within the environment associated with the medium used in this study, which would be consistent with the findings of Belenguer and colleagues, who, using YCFA + FOS medium, reported no major changes in acetate production in cocultures of *B. adolescentis* and *R. hominis* compared with that in a *B. adolescentis* monoculture [17]. However, Bhattacharya and colleagues, who cocultured *B. adolescentis* and *R. hominis* in media containing carbon sources that differed from those used in the present study, found that *R. hominis* increased butyrate production by utilizing the acetate produced by *B. adolescentis* [20]. Accordingly, these findings may indicate that acetate production could differ depending on the constituents of the growth medium used.

Based on our findings, we confirmed an increase in the production of butyrate in cocultures of BBG9‐1 and selected butyrate‐producing bacteria, which was associated with a promotion of the growth of butyrate producers. However, despite these important findings, the study has certain limitations. Notably, given that bacterial counts were not verified using quantitative PCR, we were unable to establish the degree to which coculturing promoted the growth of butyrate‐producing bacteria. Moreover, whether our findings can be replicated in vivo remains to be ascertained, thus emphasizing the necessity of further verification.

In addition to the gut itself, the gut microbiota has been widely established to play an important role in systemic health [31]. In this regard, there is growing evidence in support of the role of gut microbiota‐derived SCFAs, notably acetate, propionate, and butyrate, in the prevention, recovery, and delay of disease progression [32]. Among these SCFAs, acetate has been reported to prevent hypertension [33] and inhibit nonalcoholic fatty liver disease [34], whereas butyrate has been suggested to play an important role in the development of obesity and in the treatment of diabetes, and has also been reported to inhibit inflammation [32].

In conclusion, by coculturing BBG9‐1 and butyrate‐producing bacteria, we observed significant increases in butyrate production compared with the amounts obtained by monoculturing the respective butyrate producers. We speculate that this enhancement in the production of butyrate in cocultures could be ascribed to the utilization of BBG9‐1‐produced lactate and acetate by butyrate‐producing bacteria. Thus, by indirectly promoting butyrate production, BBG9‐1 is proposed to contribute to the maintenance of human health and disease prevention, along with an enhancement in SCFA production.

## Author Contributions

Conceptualization: Haruka Yokota and Yoshiki Tanaka. Methodology: Haruka Yokota and Yoshiki Tanaka. Formal analysis: Haruka Yokota and Yoshiki Tanaka. Data curation: Haruka Yokota and Yoshiki Tanaka. Writing – original draft preparation: Haruka Yokota. Writing – review and editing: Yoshiki Tanaka and Hiroshi Ohno. All authors have read and agreed to the published version of the manuscript.

## Conflicts of Interest

The authors declare no conflicts of interest.

## Data Availability

The data presented in this study are available on request from the corresponding author.
